# Simulation and experimental verification of ambient neutron doses in a pencil beam scanning proton therapy room as a function of treatment plan parameters

**DOI:** 10.3389/fonc.2022.903537

**Published:** 2022-09-08

**Authors:** Olivier Van Hoey, Liliana Stolarczyk, Jan Lillhök, Linda Eliasson, Natalia Mojzeszek, Malgorzata Liszka, Ali Alkhiat, Vladimir Mares, François Trompier, Sebastian Trinkl, Immaculada Martínez-Rovira, Maite Romero-Expósito, Carles Domingo, Ondrej Ploc, Roger Harrison, Pawel Olko

**Affiliations:** ^1^Belgian Nuclear Research Center (SCK CEN), Institute for Environment, Health and Safety (EHS), Mol, Belgium; ^2^Danish Centre for Particle Therapy, Aarhus University Hospital (AUH), Aarhus, Denmark; ^3^Institute of Nuclear Physics, Polish Academy of Sciences, (IFJ PAN), Krakow, Poland; ^4^The Skandion Clinic, Uppsala, Sweden; ^5^Swedish Radiation Safety Authority, Solna, Sweden; ^6^Department of Physics, Royal Institute of Technology (KTH), Stockholm, Sweden; ^7^Department of Medical Radiation Physics and Nuclear Medicine, Karolinska University Hospital, Stockholm, Sweden; ^8^Helmholtz Zentrum München, Institute of Radiation Medicine, Neuherberg, Germany; ^9^Institut de Radioprotection et de Sûreté Nucléaire (IRSN), PSE-Santé, Fontenay-aux-Roses, France; ^10^Federal Office for Radiation Protection, Neuherberg, Germany; ^11^Departament de Física, Universitat Autònoma de Barcelona, Bellaterra, Spain; ^12^Department of Radiation Dosimetry, Nuclear Physics Institute of the Czech Academy of Sciences (CAS), Prague, Czechia; ^13^Faculty of Medical Sciences, University of Newcastle upon Tyne, Newcastle Upon Tyne, United Kingdom

**Keywords:** Proton therapy, Pencil beam scanned proton therapy, Neutron doses, Monte Carlo simulations, Out-of-field neutron doses in radiation therapy, Neutron measurements

## Abstract

Out-of-field patient doses in proton therapy are dominated by neutrons. Currently, they are not taken into account by treatment planning systems. There is an increasing need to include out-of-field doses in the dose calculation, especially when treating children, pregnant patients, and patients with implants. In response to this demand, this work presents the first steps towards a tool for the prediction of out-of-field neutron doses in pencil beam scanning proton therapy facilities. As a first step, a general Monte Carlo radiation transport model for simulation of out-of-field neutron doses was set up and successfully verified by comparison of simulated and measured ambient neutron dose equivalent and neutron fluence energy spectra around a solid water phantom irradiated with a variation of different treatment plan parameters. Simulations with the verified model enabled a detailed study of the variation of the neutron ambient dose equivalent with field size, range, modulation width, use of a range shifter, and position inside the treatment room. For future work, it is planned to use this verified model to simulate out-of-field neutron doses inside the phantom and to verify the simulation results by comparison with previous in-phantom measurement campaigns. Eventually, these verified simulations will be used to build a library and a corresponding tool to allow assessment of out-of-field neutron doses at pencil beam scanning proton therapy facilities.

## 1 Introduction

One of the biggest challenges in radiotherapy is to maximize tumor damage, while sparing healthy tissues in order to minimize detrimental effects in these healthy tissues. With proton therapy, the radiation energy can be deposited more locally in the tumor in comparison with photon therapy. This leads to improved healthy tissue sparing (1). Therefore, the use of proton therapy has been increasing rapidly over the last decades with now over 100 active proton therapy facilities and over 250,000 patients treated worldwide (2).

However, despite the improved healthy tissue sparing, there is still some dose deposited in healthy tissues due to secondary and scattered radiation. The out-of-field doses in proton therapy are dominated close to the target by secondary protons and further away from the target by secondary neutrons and gamma radiation created by interactions of protons with the beamline, the patient, and the room. These out-of-field doses can lead to detrimental effects in healthy tissues and should be considered and possibly minimized during the treatment planning.

Several studies have already characterized the out-of-field neutron doses in proton therapy for some specific cases using Monte Carlo (MC) radiation transport simulations, measurements, or analytical models (3, 4). However, it is not straightforward to compare neutron doses in the literature and to estimate the neutron dose for a specific patient based on these studies due to the strong dependence of the neutron dose on the treatment plan parameters. The typical normalization of the out-of-field neutron doses to the absorbed dose in the target or the product of absorbed dose in the target and the treatment volume is not sufficient to allow direct comparison of out-of-field doses from different studies. Moreover, current treatment planning systems (TPS) do not take into account out-of-field neutron doses. However, there is an increasing need to include out-of-field neutron doses in the TPS, especially when treating children, pregnant patients, and patients with implants such as pacemakers or hearing implants.

In response to this demand, within EURADOS WG9, a dedicated task was set up. This task has the final aim to provide an easy-to-use tool to quickly assess the out-of-field neutron doses in proton therapy as a function of position with respect to the isocenter and the beam direction and the most critical treatment plan parameters such as field size, range, modulation width, use of a range shifter, and air gap between the range shifter and patient. This task focuses on active pencil beam scanning (PBS) proton therapy systems, as these systems are now becoming standard in proton therapy (2) and limit the out-of-field neutron doses by avoiding significant creation of secondary neutrons in the proton delivery system. This task will lead to a better understanding of the relation between the out-of-field neutron doses and the treatment plan parameters and allow medical physicists to evaluate and, if necessary, adapt the treatment plan also with respect to the out-of-field neutron doses and associated detrimental effects. In the end, this will contribute to improving the patient’s life expectancy and life quality.

This work presents the first steps that were performed within this task. An MC radiation transport simulation model was set up to simulate secondary radiation production and transport during patient treatment in PBS proton therapy facilities. For verification of the MC model, the neutron doses simulated outside an irradiated phantom were compared with ambient neutron monitor measurements at different positions close to the phantom for varying treatment plan parameters at two PBS proton therapy facilities. The goals of this study were to verify the MC model and to investigate the variation of the ambient neutron doses as a function of position, field size, range, modulation width, use of a range shifter, and air gap between the range shifter and phantom.

## 2 Materials and methods

### 2.1 Measurements

#### 2.1.1 Proton therapy facilities

For verification of the MC simulations, measurements were performed at two PBS proton therapy facilities. A first exploratory measurement campaign was performed at the Bronowice Cyclotron Center (CCB) Institute of Nuclear Physics (IFJ PAN) in Krakow (Poland) in May 2017. Based on the experience from this measurement campaign, a second more extensive measurement campaign was performed at the Skandion Clinic in Uppsala (Sweden) in July 2019. Both facilities are equipped with gantries with a dedicated scanning nozzle (IBA Proton Therapy System – Proteus 235). The range shifter at CCB is fixed at the nozzle, whereas at Skandion, it can be moved on the snout toward the patient.

#### 2.1.2 Experimental setup

During both measurement campaigns, rectangular target volumes were delivered from the side with the nozzle at 270° to a 30 cm × 30 cm × 60 cm solid water phantom placed on the treatment table. The isocenter was positioned at 15 cm depth in the phantom and at 15 cm from top, bottom, and the three closest side faces of the phantom. The setup was similar to that described in (5). Treatment plans were prepared using Varian Eclipse treatment planning systems (version 13.6 and 15.6 in CCB IFJ PAN and Skandion, respectively). Plan variables included field size, range, Spread Out Bragg Peak (SOBP) modulation width, use of a range shifter, and air gap between the range shifter and solid water phantom. The dose delivered to the center of the SOBP for each irradiation was 20 Gy in CCB IFJ PAN and 5 Gy in Skandion. An overview of the covered plans for both measurement campaigns is shown in **Table 1**. Data for irradiations with the range shifter are presented in this work only for the Skandion proton therapy facility. To obtain the prescribed range and modulation width, for each plan, a set of proton energies was used. Minimal and maximal proton energies are also given in **Table 1**.

**Table 1:** Overview of covered treatment plans during the measurement campaigns at the CCB IFJ PAN and Skandion proton therapy facilities with PBS.

Center	Range shifter (RS)	Air gap RS-phantom [cm]	Field size [cm^2^]	Proton energies [MeV]	Range [cm]	SOBP modulation width [cm]
CCB IFJ PAN (Krakow, Poland)	–	–	25–400	Min: 74–148 Max: 146–192	15–25	10–20
Skandion Clinic (Uppsala, Sweden)	3.1 cm WET Lexan on movable snout	5.5–23	9–625	Min: 65–103 Max: 103–212	8–25	3–25

**Figure 1:**
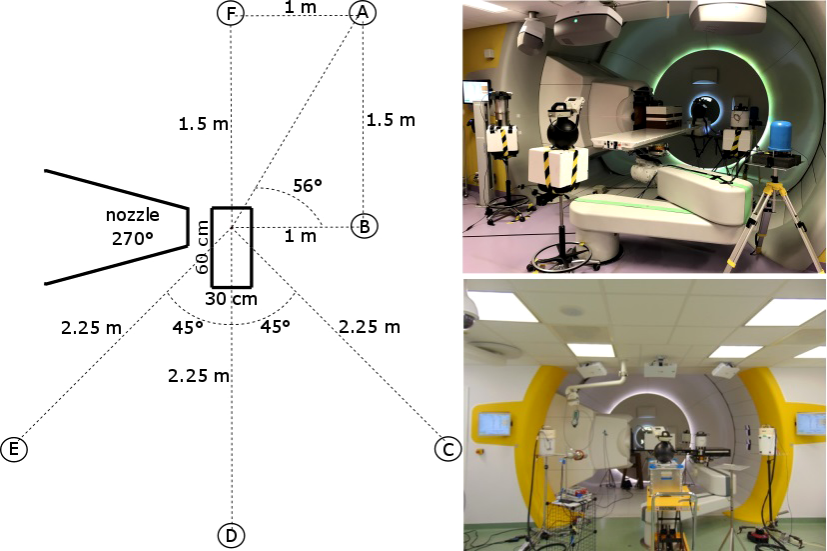
Schematic representation of the setup and the measurement positions (left), picture of the measurements at Skandion (right top), and picture of the measurements at CCB (right bottom).

Six ambient neutron monitors were positioned around the solid water phantom at six fixed positions labeled A–F. A schematic representation and pictures of the setup and the measurement positions at both facilities are shown in **Figure 1**.

#### 2.1.3 Ambient neutron monitors

The MC simulations were verified by measurements with ambient neutron monitors during the measurement campaigns at Skandion and CCB. Ambient neutron monitors measure the neutron dose in terms of the quantity ambient dose equivalent H*(10). This quantity, as defined in (6), is a measurable operational quantity that provides a conservative estimate of the radiation protection quantity effective dose. Due to limitations in experimental time and ambient neutron monitor availability, it was not possible to use the same type of ambient neutron monitor at all six positions. However, the specific aspects and uncertainties of each monitor were taken into account in the data analysis. Therefore, different types of ambient neutron monitors from the different institutes participating in the measurement campaigns were used. However, each ambient neutron monitor was kept at a fixed position throughout the whole measurement campaign. The institute and type of the ambient neutron monitors used at the six measurement positions in Skandion and CCB are listed in **Table 2**. On the second and third lines in **Table 2**, also the manufacturer and the calibration date and source are specified for each monitor. All calibrations were still valid at the time of the measurement campaigns according to the calibration procedures of the respective institutes.

**Table 2:** Ambient neutron monitors used at the different positions at Skandion and CCB.

Position	Skandion July 2019	CCB May 2017
**A**	SSM Sievert SSM, Stockholm, Sweden Calibration: 09-2018, ^252^Cf and Am-Be	CCB Wendi-II Thermo Scientific, Waltham, USA Calibration: 06-2016, ^252^Cf and Pu-Be
**B**	SCK CEN Wendi-II Thermo Scientific, Waltham, USA Calibration: 05-2018, ^252^Cf	SCK CEN Wendi-II Thermo Scientific, Waltham, USA Calibration: 11-2015, ^252^Cf
**C**	SSM Sievert SSM, Stockholm, Sweden Calibration: 09-2018, ^252^Cf and Am-Be	HMGU NM2B-495Pb NE Technology Ltd., Benham, UK Calibration: 03-2017, Am-Be
**D**	Skandion LB 6411 Berthold, Bad Wildbad, Germany Calibration: 08-2013, Am-Be	UAB LB 6411 Berthold, Bad Wildbad, Germany Calibration: 06-2010, Am-Be
**E**	IRSN HAWK Far West Technologies, Puyallup, USA Calibration: 06-2019, Am-Be	HMGU NM2B-458 NE Technology Ltd., Benham, UK Calibration: 03-2017, Am-Be
**F**	SSM LB 6411 Berthold, Bad Wildbad, Germany Calibration: 09-2018, ^252^Cf and Am-Be	NPI LB 6411 Berthold, Bad Wildbad, Germany Calibration: 10-2014, Am-Be

SSM, Swedish Radiation Safety Authority, Stockholm, Sweden.

SCK CEN, Belgian Nuclear Research Center SCK CEN, Mol, Belgium.

Skandion, Skandionkliniken, Uppsala, Sweden.

IRSN, Institut de Radioprotection et de Sûreté Nucléaire, Fontenay-aux-Roses, France.

CCB, Bronowice Cyclotron Center IFJ PAN, Krakow Poland.

HMGU, Helmholtz Zentrum Munchen, Munich, Germany.

UAB, Universitat Autònoma de Barcelona, Departament de Física, Cerdanyola del Vallès, Spain.

NPI, CAS, Nuclear Physics Institute, Prague, Czech Republic.

**Figure 2:**
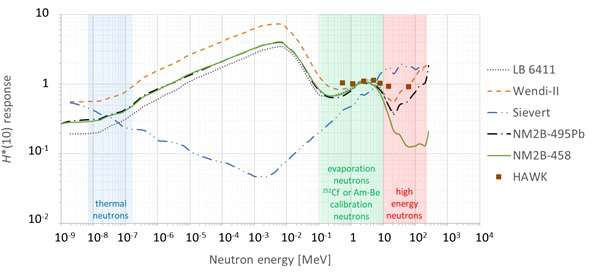
Plot with the simulated (lines) or measured (data points) H*(10) energy response functions of the different ambient neutron monitors used during the measurement campaigns at Skandion and CCB [LB 6411 (7), Wendi-II (8), Sievert [this work], NM2B-495Pb and NM2B-458 (9), HAWK (10)].

A very important characteristic of ambient neutron monitors is their energy response in terms of H*(10). Ideally, the H*(10) energy response should be close to unity for all possible neutron energies. However, in practice, no existing ambient neutron monitor has a perfect energy response for the wide range of possible neutron energies. This has to be taken into account when analyzing the measurement results and comparing them with the MC simulations. The different ambient neutron monitors are described in detail below. The H*(10) energy response functions are compared in **Figure 2**. Where necessary, they were normalized taking into account the energy spectrum of ^252^Cf or Am-Be neutrons used during the calibration. For HAWK, these response data are obtained from measurements, while for the other monitors, the response data are obtained from simulations.

Neutron energy spectra at proton therapy facilities typically exhibit peaks for thermal, evaporation, and high-energy neutrons (11). The energy spectra of ^252^Cf or Am-Be neutrons fall in the same energy range as the evaporation neutrons. The energy ranges of these peaks are indicated in **Figure 2**.

**Figure 2** shows that the energy response is close to unity for all monitors in the evaporation neutron energy range. This is expected because they are calibrated with ^252^Cf or Am-Be neutrons in the same energy range. In the other energy ranges there are significant deviations from unity and also significant differences between the different monitors.

One can see in **Figure 2** that in the thermal energy range, the ambient neutron monitors exhibit an under-response between a factor of two and five. It can also be seen in **Figure 2** that conventional ambient neutron monitors such as LB 6411, Wendi-II, NM2B-495Pb, and NM2B-458 exhibit a typical over-response in the epithermal energy range related to the very strong thermalization of epithermal neutrons in their moderator sphere. On the other hand, Tissue Equivalent Proportional Counters (TEPCs) such as Sievert have an under-response in the epithermal energy range due to lower backscattering in the gas volume in comparison with the ICRU sphere in the definition of H*(10). A similar under-response is expected for HAWK, but no response data are available in the epithermal energy range. However, neutrons in the thermal and epithermal energy range are not expected to contribute more than a few percent to the neutron H*(10) in proton therapy treatment rooms (12). Therefore, the under-response in the thermal neutron range and the over-response and under-response of respectively conventional ambient neutron monitors and TEPCs in the epithermal neutron range are not expected to affect the measurements significantly.

Finally, one can observe in **Figure 2** that the H*(10) energy response of LB 6411 and NM2B-458 drops to zero above 10–20 MeV due to their very limited sensitivity for high-energy neutrons. The sensitivity is increased for Wendi-II and NM2B-495Pb by an additional metallic shield embedded in the moderator. However, even for these monitors, there is still a limited under-response for high-energy neutrons. TEPCs such as Sievert and HAWK are intrinsically sensitive for high-energy neutrons. However, for Sievert, one can see that there is a limited over-response for high-energy neutrons. As high-energy neutrons can deliver a large fraction of the neutron dose in proton therapy rooms (12), the under-response and over-response of the ambient neutron monitors for high-energy neutrons is expected to affect the measurement results significantly. This will be discussed in detail in Sections 3.1.1.4 and 3.2.2.

##### 2.1.3.1 Sievert

The two Sievert instruments used by SSM are TEPCs, made of A-150 plastic and filled with a propane-based tissue-equivalent gas held at 1.37 kPa pressure, simulating a tissue volume with a mean chord length of 1.88 µm. The sensitive volume is 1.207 dm^3^, with 11.5 cm for both height and diameter. The electric charge is measured using a capacitor feedback electrometer, where the voltage over a 1-nF capacitor is measured over a charge collection time of 0.1 s (13).

The average absorbed dose during a charge collection time is given by


$${\bar{D}}_{det}=\;\frac{\bar{q}W/e}{M\;m_{det}}$$

where $\bar{q}$ is the mean collected charge during the time interval, W/e is the average energy required to create an ion pair, M is the gas multiplication factor, and $m_{det}$ the mass of the detector gas mass

The dose-mean lineal energy is calculated using the variance method


$${\bar{y}}_{D}=\;\frac{m_{det}\;V_{D,rel}\;}{\bar{l}}{\bar{D}}_{det}\;$$

where *V*_*D*,*rel*_  is the relative variance in the absorbed dose during repeated charge integrations and $\bar{l}$ is the mean chord length of the simulated tissue volume

The dose equivalent H* is in turn determined by


$$H^{*}\;=\;{\bar{D}}_{det}\;\left(a+b{\bar{y}}_{D}\right)$$

where the constants *a* = 0.88 and *b* = 0.09 µm/keV are chosen for the typical high-energy neutrons present in the proton therapy rooms (13). Thus, by measuring the dose-mean lineal energy using the TEPC, the dose equivalent in a mixed radiation field can be determined.

The absorbed dose fractions due to low and high LET radiation are calculated from the measured ${\bar{y}}_{D}$ value and the dose-mean lineal energies of photon and neutron components, that is,


$${\bar{y}}_{D}=\;{\bar{y}}_{D,\gamma }d_{\gamma }+\;{\bar{y}}_{D,n}d_{n}=\;{\bar{y}}_{D,\gamma }d_{\gamma }+\;{\bar{y}}_{D,n}\left(1-d_{\gamma }\right)$$

The dose-mean lineal energies for photons ${\bar{y}}_{D,\gamma }=1.4\;\text{keV}/\text{µm}$ and neutrons ${\bar{y}}_{D,n}=96\;\text{keV}/\text{µm}$ are calculated from MC-simulated relative contribution in the mixed field. From the relative dose contributions, the dose equivalent for photons and neutrons, $H_{\gamma }^{*}$ and $H_{n}^{*}$ are estimated from

$H_{n}^{*}=d_{n}D_{det}\left(a+b{\bar{y}}_{D,n}\right)$ and


$$H_{\gamma }^{*}=(1-d_{n})D_{det}\left(a+b{\bar{y}}_{D,\gamma }\right)$$

Using the relative dose fractions, a value of 28 eV was obtained for W/e.

In this work, single spot measurements at proton beam energies of 70, 146, and 212 MeV were performed to calculate ${\bar{y}}_{D}$ values for different energies and positions. To determine the dose equivalent for the scanned irradiations, the maximum proton energy was used to choose which ${\bar{y}}_{D}$ value to use. No ${\bar{y}}_{D}$ measurements were performed with a range shifter. Therefore, no H*(10) measurements for the irradiations with the range shifter are presented for positions A and C at Skandion where the Sievert was used at those positions.

The Sievert H*(10) energy response plotted in **Figure 2** was calculated by means of MC simulations. The response is fairly close to unity over the whole energy range, except for an under-response for epithermal neutrons and a slight over-response for high-energy neutrons.

##### 2.1.3.2 HAWK

The HAWK environmental Monitoring System FW-AD1 from Far West Technology Inc. used by IRSN is a microdosimetric single-event TEPC-system. The detector is spherical with 127 mm diameter (Benjamin type) and filled with pure propane gas at 933 Pa to simulate an energy deposition in 2 µm biological site and a mean chord length of 1.33 µm (14). HAWK measures the energy deposition spectrum from particles correlated to a single initial particle event on a lineal energy scale calibrated using a proton edge calibration. The absorbed dose distribution in lineal energy *d*(*y*) and the low- and high-LET components are defined as the contributions below and above 10 keV/µm respectively. From this distribution, the dose equivalent is calculated according to


$$H^{*}=H_{low}^{*}+H_{high}^{*}=N_{low}\int_{0.5}^{10}Q\left(y\right)d\left(y\right)dy+N_{high}\int_{10}^{1024}Q\left(y\right)d\left(y\right)dy$$

Here, *N*_low_ and *N*_high_ are the low-LET and high-LET H*(10)-correction calibration factors from ^137^Cs and ^60^Co photon and Am-Be neutron fields (10, 15). *N*_low_ aims to compensate for the relatively high value of the electronic threshold, making it impossible to measure events below 0.5 keV/µm. *N*_high_ aims to compensate for the uncorrected y value for the proton edge set by the manufacturer for the y scale calibration. *N*_low_ is equal to 1.1 ± 0.02 and *N*_high_ to 0.8 ± 0.09. The high-LET component of the dose equivalent $H_{high}^{*}$ is used in this work as an approximation of the neutron H*(10).

For the H*(10) energy response of the HAWK, only a limited number of data points in the energy range from 0.5 to 60 MeV are available from (10). These are also shown in **Figure 2**. It can be seen that the response is very close to unity in this energy range. Similar to the Sievert and other TEPCs, also for the HAWK, an under-response for energies below 0.5 MeV can be expected. For high-energy neutrons, the response is expected to be close to unity as well.

##### 2.1.3.3 LB 6411

The LB 6411 ambient neutron monitor from Berthold Technologies used by SSM, UAB, and NPI consists of a 25-cm-diameter polyethylene moderator sphere with internal Cd absorbers and perforations that surrounds a cylindrical ^3^He proportional counter (7). The neutron sensitivity is around 3 counts per nSv of neutron H*(10). It has excellent photon rejection capabilities with less than 30 µSv/h of photon response in a 10 mSv/h photon radiation field.

This ambient neutron monitor is designed to measure thermal to 20 MeV neutrons and it is known to have a strongly decreasing sensitivity to neutrons above 20 MeV. This is clearly reflected in the H*(10) energy response plotted in **Figure 2**, as obtained from MC simulations (7). The response is fairly close to unity over the whole energy range, except for an over-response in the epithermal energy range and a strong under-response for neutrons with energies above about 20 MeV for which the LB 6411 is almost insensitive.

##### 2.1.3.4 Wendi-II

The Wendi-II ambient neutron monitor from Thermo Scientific used by SCK CEN and CCB is an extended-range ambient neutron monitor designed by Olsher et al. (16). It consists of a cylindrical polyethylene moderator with an inner tungsten shell that surrounds a cylindrical ^3^He proportional counter. The neutron sensitivity is around 3 counts per nSv of neutron H*(10). It has excellent photon rejection capabilities with less than 5 µSv/h of photon response in a 100 mSv/h photon radiation field.

The tungsten shell embedded in the polyethylene moderator greatly enhances the Wendi-II response to high-energy neutrons, extending the measurement range to about 5 GeV and thus well beyond the maximum neutron energy of about 200 MeV encountered in proton therapy facilities. This is clearly reflected in the H*(10) energy response plotted in **Figure 2**, as obtained from MCNPX 2.7 simulations (8). The response is fairly close to unity over the whole energy range, except for an over-response for epithermal neutrons.

##### 2.1.3.5 NM2B-458 and NM2B-495Pb

A conventional NM2B‐458 and an extended‐range NM2B‐495Pb ambient neutron monitor were used by HMGU. These monitors are based on the Andersson-Braun (AB) model and manufactured by NE Technology Ltd. They consist of a cylindrical BF_3_ proportional counters of 3.1 cm diameter and 7.2 cm active length surrounded by an inner 1.7-cm-thick moderating polyethylene layer, a 0.6-cm-thick boron‐doped synthetic rubber absorber, and an outer 6.9-cm-thick polyethylene moderator. The NM2B‐495Pb ambient neutron monitor additionally has a 1 cm thick lead shell surrounding the boron rubber to increase the response to high-energy neutrons.

The H*(10) energy responses of both ambient neutron monitors were calculated by means of MC simulations in the energy range from thermal to 10 GeV (9) and are shown in **Figure 2**. The response is fairly close to unity over the whole energy range up to about 10 MeV for both ambient neutron monitors, except for an over-response for epithermal neutrons. For higher-energy neutrons, only the NM2B-495Pb has a response fairly close to unity due to the additional lead shell. For the NM2B-458, the response for high-energy neutrons drops very rapidly above about 10 MeV.

### 2.2 Simulations

All the simulations in this work were performed with the MC radiation transport code MCNP6.2 (17).

**Figure 3:**
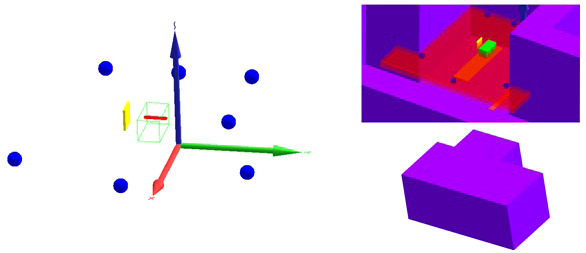
Geometrical models implemented in MCNP6.2. (Left) Model without room: solid water phantom (green), SOBP mesh tally (red), range shifter (yellow), and detectors (blue). (Right) Model with room with a view through the roof inside the room (top) and a view of the outside of the room (bottom): solid water phantom (green), positioning uncertainty mesh tally (red), range shifter (yellow), detectors (blue), table (orange), and walls, ceiling, and floor (purple).

The first important input in these simulations is the geometrical model. Two different geometrical models were implemented in this work. Firstly, a model was developed that does not take into account the specifics of the room. This model is shown on the left in **Figure 3**. It consists of

* the 60-cm-long and 30 cm by 30 cm cross section solid water phantom made of white polystyrene type RW3 with 2% by weight TiO_2_ and a density of 1.03 g/cm³ (green);
* a mesh tally inside the phantom to tally the SOBP delivered by the proton beam (red);
* the 3.11-cm-thick and 30 cm by 30 cm cross section range shifter made of Lexan with a density of 1.2 g/cm³ positioned with the appropriate air gap with respect to the phantom edge for the cases in which a range shifter was used (yellow); and
* six 20-cm-diameter spherical air cells at the detector positions for tallying the neutron fluence energy spectrum and neutron H*(10) (blue).

The advantage of this model is that it is generally valid for any PBS proton therapy facility after adaptation of the range shifter dimensions. The disadvantage is that without inclusion of the walls, floor, ceiling, patient table, gantry cylinders, gantry cone, counterweight, and other components in the room, the neutrons created inside these components are not taken into account. The scattered thermal and epithermal neutrons are not expected to contribute more than a few percent to the total neutron H*(10) (12). However, evaporation neutrons that are partly created in iron-rich components such as the gantry cylinders, the gantry cone, and the counterweight (18) can contribute up to about 50% of the total neutron H*(10) (19).

As the goal of this work is to develop a tool to predict out-of-field neutron doses in any PBS proton therapy facility and to get a better understanding of the trends of the out-of-field neutron doses as a function of treatment plan parameters, development of detailed room models of the Skandion and CCB proton therapy facilities was beyond the scope of this work. However, in order to better understand the potential sources of deviations between simulations and measurements, it was decided to also develop a second geometrical model for the Skandion facility with a simplified representation of the room. This model is shown on the right in **Figure 3**. The top picture shows a view inside the room through the roof, while the bottom picture shows an outside view of the room. In addition to the components implemented in the first model, this second model also contains

* the 6-cm-thick and 220 cm by 52 cm table made of Kevlar with a density of 1.44 g/cm³ (orange);
* walls, ceiling, and floor made of 1-m-thick ordinary NIST concrete (20) with a density of 2.3 g/cm³ (purple); and
* a mesh tally for assessing the positioning uncertainty of the measurements (red).

The 1-m-thick concrete everywhere is an approximation and might lead to an overestimation of the scattered neutrons in the gantry area. Also, the iron-rich components of the gantry are not taken into account. This might lead to an underestimation of the evaporation neutrons that are, to a great extent, created in these components (18).

The second meaningful input in the simulations is the definition of the radiation source. The rectangular proton fields were modeled as a monodirectional collimated uniform square proton beam with size corresponding to the field size. The proton energy is sampled from the superposition of Gaussian energy distributions with $\sqrt{2}\sigma =1.5\%$ and energies and weights obtained from the layer information in the *.pld file from the TPS.

The neutron reaction cross sections were taken from the ENDF/B-VIII.0 database (21) for a temperature of 0.02585 eV. Both protons and neutrons were tracked with a high-energy cutoff at 240 MeV, well above the maximum proton energy of 212 MeV. The Bertini Dresner intranuclear cascade and evaporation model was selected because it gave reliable results in previous simulations of neutron doses in proton therapy facilities (4, 22). Furthermore, a limited sensitivity study showed less than 10% difference in simulated neutron H*(10) values when using the default cross sections and physics models in MCNP6.2.

Finally, it is necessary to define the appropriate tallies in the simulations in order to obtain the desired quantities. A type 3 volumetric energy deposition mesh tally was defined in the target volume of the solid water phantom for assessing the SOBP absorbed dose profile. In this way, it could be checked whether the proton beam was modeled in a sufficiently realistic manner. F4 fluence energy spectrum and F4 H*(10) tallies were defined in the detector cells. The energy bin width in lethargy was fixed at $ln\left(\frac{Ei}{E_{i-1}}\right)=0.26$ . This allowed direct comparison with the neutron H*(10) values measured by the ambient neutron monitors in this work and the neutron energy spectra measured with Bonner spheres during a previous measurement campaign at a similar PBS proton therapy facility in Trento (Italy) (11). A type 1 dose mesh tally with ICRP 74 fluence to H*(10) conversion coefficients using the TMESH DOSE keyword with option ic = 40 was defined in the horizontal plane at the height of the detectors to obtain an estimation of the positioning uncertainty of the measurements. The simulation results are all expressed per simulation particle. However, as also the SOBP absorbed dose profile is simulated, all simulation results can be divided by the absorbed dose in the center of the SOBP per simulation particle in order to obtain the results expressed per unit of absorbed dose in the center of the SOBP. This allows direct quantitative comparison between the simulations and measurements.

## 3 Results and discussion

### 3.1 Uncertainties

For clarity of the plots presented here, uncertainty bars were not always added to the measurement and simulation results and their ratios. However, the order of magnitude of the uncertainties and the different contributions to the uncertainties are discussed in detail in this subsection. All uncertainties presented here are expressed at the *k* = 1 level.

#### 3.1.1 Measurements

##### 3.1.1.1 Positioning uncertainty

The positioning uncertainty is estimated by assuming that the positioning of the detectors was done with a precision of about 5 cm. The associated uncertainty on the measured H*(10) was estimated based on the simulated type 1 dose mesh tally with ICRP 74 fluence to H*(10) conversion coefficients in the horizontal plane at the height of the detectors. The simulated H*(10) on the mesh tally was averaged over the volume of a detector, shifting the detector positions 5 cm from the reference positions in the positive and negative direction along the *x*, *y*, and *z* axes. The positioning uncertainty was then assessed assuming a uniform distribution between the lowest and highest dose values obtained for each detector position. The obtained uncertainties averaged over all treatment plan parameters vary between 2.6% for position A and 5.0% for position B with a global average of 4%. As expected, a higher positioning uncertainty is found for the detector positions closer to the isocenter due to the higher dose gradient.

##### 3.1.1.2 Calibration and instrument specific uncertainty

All ambient neutron monitors were calibrated using ^252^Cf or Am-Be neutron sources. The calibration and instrument-specific uncertainty is estimated to be 5% for Sievert, 6% for HAWK, and about 2% for the other ambient neutron monitors.

##### 3.1.1.3 Statistical uncertainty

The statistical uncertainty is the uncertainty on the measurement itself related to Poisson counting statistics. The relative statistical uncertainty was calculated as the inverse square root of the number of counts obtained during the measurement. This uncertainty ranges between 6% and 18% with an average of 10% for Sievert at position A, between 9% and 25% with an average of 20% for Sievert at position C, between 1% and 3% for HAWK and well below 1% for the other ambient neutron monitors in most cases with exceptions up to 2% for very low doses.

##### 3.1.1.4 Energy response uncertainty

The energy response uncertainty is related to the imperfect H*(10) energy response of the ambient neutron monitors. The expected responses of the different ambient neutron monitors at their measurement positions were estimated by convoluting the neutron H*(10) energy spectra simulated with the model without the room specifications at their measurement position with the monitor H*(10) energy response. The minimum, maximum, and average H*(10) responses for the different treatment plan parameters for each monitor are shown in **Table 3**.

**Table 3:** Minimum, maximum, and average H*(10) responses of the different ambient neutron monitors at the different positions A-F as estimated from the convolution of the neutron H*(10) energy spectra simulated with the model without room specifications and the simulated H*(10) energy responses.

Position	Skandion				CCB			
	Detector	Min	Max	Average	Detector	Min	Max	Average
**A**	**SSM Sievert**	1.34	1.42	1.38	**IFJ Wendi-II**	0.78	0.86	0.81
**B**	**SCK CEN Wendi-II**	0.72	0.97	0.85	**SCK CEN Wendi-II**	0.78	0.99	0.88
**C**	**SSM Sievert**	1.40	1.47	1.45	**HMGU NM2B-495Pb**	0.59	0.66	0.62
**D**	**Skandion LB 6411**	0.33	0.61	0.46	**UAB LB 6411**	0.35	0.53	0.44
**E**	**IRSN HAWK**	–	–	–	**HMGU NM2B-458**	0.81	0.90	0.86
**F**	**SSM LB 6411**	0.58	0.72	0.66	**NPI LB 6411**	0.63	0.73	0.69

No data are shown for HAWK because the H*(10) energy response was only available for a limited energy range. However, as discussed in Section 2.1.3.2, its response is expected to be close to unity for the measurements in this work. The NM2B-495Pb with extended energy range seems to perform worse than the NM2B-458 without extended energy range. However, it has to be taken into account that the NM2B-495Pb was used at position C, which is in the forward direction where high-energy neutrons are expected to contribute significantly to the dose, whereas the NM2B-458 was used at position E in the backward direction where virtually no high-energy neutrons are expected.

It can be seen that significant overestimations up to 47% and significant underestimations up to 67% are possible. Hence, the energy response will often be the biggest source of measurement uncertainty. Sievert shows a systematic overestimation, while all other ambient neutron monitors exhibit a systematic underestimation. This is well in line with the over-response and under-response for high-energy neutrons for respectively the Sievert and the other ambient neutron monitors as shown in **Figure 2** and discussed in Section 2.1.3.

##### 3.1.1.5 Target dose uncertainty

Finally, the uncertainty on the delivered absorbed dose in the target volume also contributes to the measurement uncertainty as both measurements and simulations are normalized to the absorbed dose in the target volume. This uncertainty is estimated to be about 2.5%. This uncertainty also includes the day-to-day variations of less than 0.5% based on the daily QA measurements.

##### 3.1.1.6 Combined uncertainty

From the discussion above, it is clear that the measurement uncertainty varies significantly. It depends on the type of ambient neutron monitor, the measurement position, and the treatment plan parameters. Combining all the above uncertainties, it can be estimated that the combined measurement uncertainty is typically in the range between 15% and 30%.

#### 3.1.2 Simulations

The number of particles in the MCNP6.2 simulations was taken sufficiently high to keep the statistical uncertainties on the simulated H*(10) values below 1% and on the simulated fluence energy spectra below 5% for all energy bins contributing significantly to the total fluence. However, the statistical uncertainties of MC simulations are only a minor component of the total uncertainty. The total uncertainty is dominated by uncertainties in the reaction cross sections, uncertainties in the physics models and simplifications or inaccuracies in the model geometry. Assessment of these uncertainties is not straightforward and was considered beyond the scope of this work. It just has to be kept in mind when comparing the measurements and simulations that the simulation results come with a significant uncertainty as well.

### 3.2 Verification of the simulations

#### 3.2.1 SOBP depth profile

**Figure 4:**
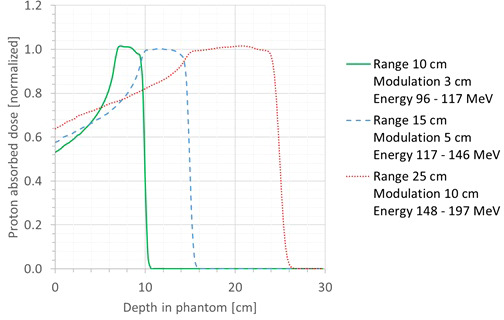
Plot of the simulated SOBP profiles for three different cases: range, 10 cm; modulation width, 3 cm (green full line); range, 15 cm; modulation width, 5 cm (blue dashed line); range, 25 cm; modulation width, 10 cm (red dotted line).

First, it was checked whether the proton beam was modeled in a sufficiently realistic way. For this, the simulated SOBP profiles were plotted and compared with the range and modulation width of the corresponding treatment plan. The shape of the SOBP profiles agreed very well with the expected shape based on the range and modulation width for all cases. Three representative cases are shown in **Figure 4**. In this plot, the SOBP profiles are normalized to the average of the plateau of the SOBP. Hence, it could be concluded that the proton beam was modeled in a sufficiently realistic way.

#### 3.2.2 Neutron ambient dose equivalent

Further verification of the simulation model was performed by comparison of the neutron ambient dose equivalent H*(10) from the simulations with the measured H*(10) from the ambient neutron monitors. The first measurement campaign was performed at CCB for a limited set of treatment plan parameters in May 2017. Later, in July 2019, a more extensive measurement campaign was performed at Skandion. The comparison between simulations and measurements will be made here in detail for the Skandion measurement campaign as it provides the most extensive data set. Furthermore, an approximate model of the Skandion treatment room was implemented in the simulations as explained in Section 2.2. In Section 3.3, the variation of the H*(10) with position and treatment plan parameters, and the measurements at Skandion and CCB are also compared with each other. An overview of all the irradiations and their treatment plan parameters for the measurement campaigns performed at Skandion and CCB is shown in **Tables A.1** and **A.2** in the appendix. Each of the listed irradiations was performed only once due to beam time limitations and expected stability of the irradiations.

**Figure 5:**
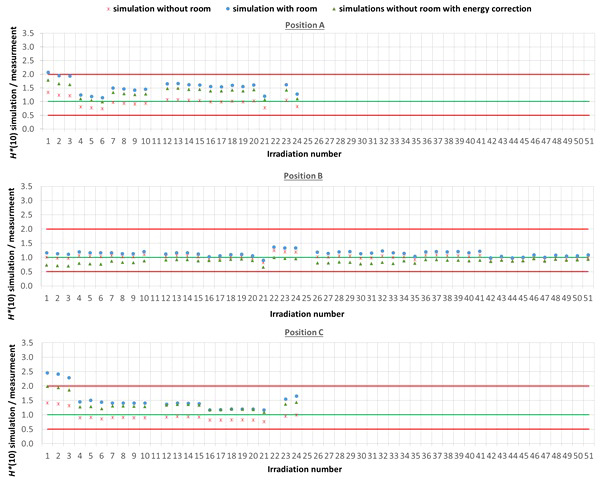
Plots with the ratio of simulated over measured H*(10) for positions (A–C) for the Skandion measurement campaign. Three cases are shown: simulations without room (red crosses), simulations with room (blue circles) and simulations without room and correction for the imperfect ambient neutron monitor H*(10) energy response (green triangles). A green line indicating a ratio of one and two red lines indicating a factor of two over- or under-response are added to guide the eye.

**Figure 6:**
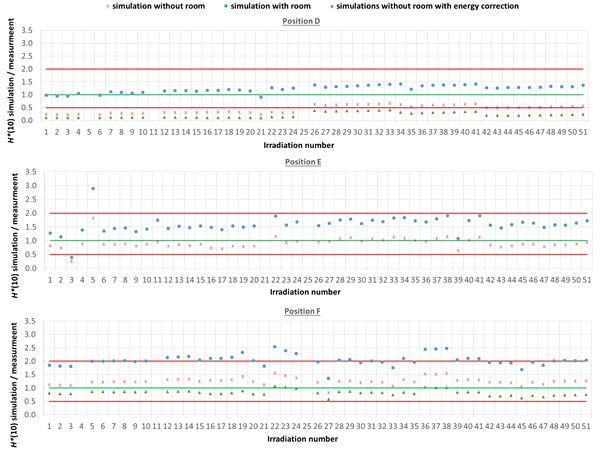
Plots with the ratio of simulated over measured H*(10) for positions (D–F) for the Skandion measurement campaign. Three cases are shown: simulations without room (red crosses), simulations with room (blue circles), and simulations without room and correction for the imperfect ambient neutron monitor H*(10) energy response (green triangles). A green line indicating a ratio of one and two red lines indicating a factor of two over- or under-response are added to guide the eye.

The comparison between simulated and measured H*(10) is made for three different cases. In the first case, the measured H*(10) is compared with the simulated H*(10) from the simulations with the general geometrical model not taking into account the specifics of the room. In order to evaluate the uncertainty related to disregarding the specifics of the room, in the second case, the measured H*(10) is compared with the simulated H*(10) from the simulations with the simplified Skandion room model. For assessing the uncertainty related to the imperfect ambient neutron monitor H*(10) energy response, in the third case, the measured H*(10) is compared with the H*(10) obtained by convolution of the simulated neutron H*(10) energy spectra from the simulations without the specifics of the room with the H*(10) energy response of the appropriate ambient neutron monitor. For position E, the third case is not applicable, because the H*(10) energy response of the HAWK used at this position is only available for a limited energy range. **Table 4** gives an overview of the average ratio of simulated over measured H*(10) per position and averaged over all positions for the three cases. The separate ratios of simulated over measured H*(10) for all irradiations separately are plotted in **Figure 5** (position A, B, and C) and in **Figure 6** (position D, E, and F) as a function of the irradiation number as specified in **Table A.1** in the appendix. The ratios for the simulations without specifics of the room are shown as red crosses, the ratios for the simulations with specifics of the room are shown as blue circles, and the ratios for the simulations without specifics of the room and correction for the imperfect ambient neutron monitor H*(10) energy response are shown as green triangles. A green line indicating a ratio of one and two red lines indicating a factor of two over- or under-response are added to guide the eye. Error bars are not added here for clarity of the plots. However, a more detailed comparison with error bars is presented in Sections 3.3.2–3.3.5.

**Table 4:** Overview of the average ratio of simulated over measured H*(10) per position and averaged over all positions for the Skandion measurement campaign.

H*(10) Simulation/measurement	A	B	C	D	E	F	Average
Simulation without room	0.99	1.01	0.95	0.44	0.92	1.26	0.92
Simulation with room	1.54	1.13	1.50	1.23	1.58	2.04	1.50
Simulation without room with energy correction	1.36	0.86	1.37	0.21	–	0.82	1.36

One can see in **Figures 5**, **6** that even without the room in the simulation model and without energy correction, most of the simulation data are already within a factor of two from the measurements. Only for position D and for one irradiation at position E are there stronger underestimations. From the results in **Table 4** and **Figures 5**, **6**, one can also see that introduction of the room in the simulation model significantly increases the simulated H*(10) values. This systematic increase for all irradiations is caused by the additional scattered neutrons from the room contributing to the neutron dose. For positions A, C, E, and F, the increase is about 60%. For position D, a much higher increase with almost a factor of three is observed. This is caused by the fact that position D is the most distant position from the gantry area perpendicular to the beam direction, where scattered neutrons are expected to contribute the most. There might also be an important dose contribution from neutrons created inside the bending magnet of the gantry, which are not taken into account in the simulations. These additional scattered neutrons are probably the reason for the underestimation of the neutron dose at position D in the simulations. On the other hand, for position B, the increase is limited to only 12%. This is also expected because position B is the position closest to the isocenter at only 1 m in the direction of the beam, where scattered neutrons are not expected to contribute significantly.

From the results in **Table 4** and **Figures 5** and **6**, one can also see that when correcting for the imperfect energy response of the ambient neutron monitors, the ratio of simulations over measurements systematically increases with about 40% for the Sievert measurements at positions A and C, while it systematically decreases about 15%, 50%, and 35% for respectively Wendi-II at position B and LB 6411 at positions D and F. This is related to the imperfect energy response of the ambient monitors for high-energy neutrons as discussed in Sections 2.1.3 and 3.1.1.4.

For positions A, B, C, and E, the average agreement between the measurements and the simulations without the specifics of the room is within 8%. The agreement for individual irradiations is also well within 30% for most irradiations. Taking into account the measurement uncertainty in the range of 15% to 30% and the significant uncertainty on the simulations, this can already be considered as good agreement. The H*(10) values from the simulations with the specifics of the room in **Table 4** show a significant overestimation with respect to the measurements. This overestimation is probably caused by an overestimation of the scattered neutron dose contribution by using 1-m-thick concrete everywhere in the gantry area in the simulation model. Furthermore, there are also uncertainties in the concrete composition and concrete hydrogen content that can both strongly affect the scattered neutron contribution. Correction for the imperfect energy response of the ambient neutron monitors leads to a decrease of the measurements for positions A and C and an increase for position B as expected from the responses tabulated in **Table 3**. Correction for the imperfect energy response worsens the agreement between measurements and simulations. This can be caused by uncertainties in the neutron monitor energy response functions, uncertainties in the simulated neutron fluence energy spectra due to absence of the room and other important components influencing the energy spectra in the simulations, and the strong sensitivity of the neutron monitor response on the neutron energy. These observed effects of scattered neutrons from the room and the imperfect energy response of the ambient neutron monitor can also account for the limited deviations found between the measurements and the simulations.

For position D, one can observe a systematic underestimation of 56% for the simulations without the specifics of the room in comparison with the measurements. This underestimation could be expected because position D is 2.25 m away from the isocenter in a direction perpendicular to the proton beam. There, neutrons scattered by the room are expected to contribute significantly to the neutron dose. This is confirmed by the fact that the simulated H*(10) values for position D for the simulations with the specifics of the room are significantly higher and on average 23% above the measured H*(10) values. This overestimation can be related to uncertainties in the concrete composition and concrete hydrogen content, which can both strongly affect the scattered neutron contribution. Also, the systematic under-response of the LB 6411 ambient neutron monitor could explain this overestimation as can be seen in **Table 3** and from the simulation results without the specifics of the room with energy correction in **Table 4**.

For position F, it can be seen that there is a systematic overestimation of 26% for the simulations without the specifics of the room in comparison with the measurements. Adding the specifics of the room in the simulations worsens the overestimation to about a factor of two. This is probably again due to an overestimation of the scattered neutron dose contribution because of the use of 1-m-thick concrete everywhere in the gantry area in the simulation model, especially since position F is completely at the back in the gantry area. The overestimation is probably caused by the underestimation of the LB 6411 ambient neutron monitor as can be seen from **Table 3** and from the 30% decrease of the simulated H*(10) values when applying the energy correction in **Table 4**.

It can be concluded from this comparison that the simulation model without specifics of the room is sufficiently realistic. The deviations between the measured and simulated neutron H*(10) values were within the estimated combined uncertainty for all positions except for position D further away from the isocenter and perpendicular to the beam direction where scattered neutrons form an important dose contribution. Therefore, it is expected that also for the simulation of out-of-field neutron doses within the phantom, this simulation model will perform with sufficiently good accuracy.

#### 3.2.3 Neutron fluence energy spectrum

The neutron fluence energy spectra are very important as both the neutron fluence to dose equivalent conversion coefficient and the ambient neutron monitor H*(10) energy responses depend strongly on the neutron energy. Therefore, verification of the simulated neutron fluence energy spectra was also performed by comparison with Bonner sphere measurements performed at a previous measurement campaign at a very similar proton therapy facility in Trento (11). The setup was very similar to the measurement campaigns at Skandion and CCB. The measurements in Trento were performed during an irradiation with 20 cm range, 10 cm modulation width, and 10 cm × 10 cm field size without a range shifter. The only difference was that position B in Trento was at a distance of 1.5 m from the isocenter instead of 1 m at Skandion and CCB.

**Figure 7:**
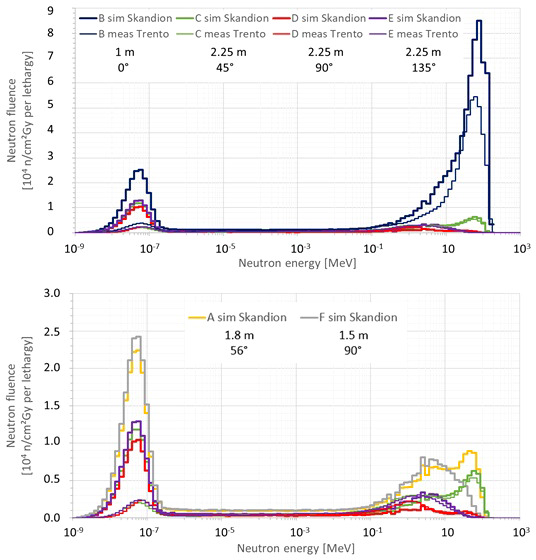
Plots comparing the neutron fluence energy spectra at positions (B–E) simulated in this work for Skandion (thick lines) and the neutron fluence energy spectra measured with Bonner spheres at the equivalent positions in Trento (thin lines). The treatment plan parameters were the same in both data sets: 20 cm range, 10 cm modulation width and 10 cm × 10 cm field size without a range shifter. The fluence energy spectra are given per unit of absorbed dose in the target in Gy and plotted per unit lethargy. The top plot shows all positions, while the bottom plot zooms in on the positions (C–E) to have a more detailed view. The bottom plot additionally shows the neutron fluence energy spectra at positions A and F simulated in this work for Skandion for comparison.

**Figure 7** compares the neutron fluence energy spectra at positions B, C, D, and E simulated in this work for Skandion with the simulation model with the specifics of the room (thick lines) and the neutron fluence energy spectra measured with Bonner spheres at the equivalent positions in Trento (thin lines). The treatment plan parameters were the same in both data sets: 20 cm range, 10 cm modulation width, and 10 cm × 10 cm field size without a range shifter. The fluence energy spectra are given per unit of absorbed dose in the target in Gy and plotted per unit lethargy. The neutron lethargy is defined as $ln\left(\frac{E_{i}}{E_{i-1}}\right)=0.26$ with *E*_*i*_ and *E*_*i*−1_ being the upper and lower energies of the energy bin, respectively. Neutron fluence energy spectra are commonly plotted per unit lethargy when the energy axis is logarithmic because, in this way, equal areas under the spectra represent equal amount of fluence. The top plot shows all positions, while the bottom plot zooms in on positions C, D, and E to have a more detailed view. The bottom plot additionally shows the neutron fluence energy spectra at positions A and F simulated in this work for Skandion with the simulation model with the specifics of the room for comparison.

Very good agreement between simulations and measurements is observed for the high-energy neutron peak. Only for position B is a significantly higher peak observed in the simulations as expected because position B is 50 cm closer to the isocenter in the simulations in comparison with the measurements in Trento.

The evaporation neutron peaks for positions C and D are higher in the measurements in Trento than in the simulations. For position D, the shape is also different. This is probably caused by simplifications in the room model or differences in the rooms of Skandion and Trento. Evaporation neutrons are partly created in iron-rich components such as the gantry cylinders, the gantry cone, and the counterweight (18), and these components were not taken into account in the simplified room model of the simulations. However, the contribution of this peak to the total dose is less important closer to the phantom and inside the phantom as demonstrated by the good agreement between measured and simulated H*(10) values in Section 3.2.2.

The thermal neutron peaks in the simulations are systematically higher than those of the measurements in Trento. This is probably again caused by simplifications in the room model in the simulations or differences between the rooms in Skandion and Trento. As discussed already in Section 3.2.2, the 1-m-thick concrete everywhere probably overestimates the scattered neutron contribution and thus also the thermal neutron peak. Anyhow, thermal and even epithermal neutrons are expected to contribute only to the maximum, a few percent of the total out-of-field neutron dose in proton therapy (12).

In Section 3.2.2, it was shown that the use of the simulated neutron fluence energy spectra to correct for the imperfect energy response of the ambient neutron monitors can explain some of the deviations between the simulated and the measured H*(10) values. This gives further confidence that the simulated neutron fluence energy spectra are sufficiently realistic.

### 3.3 Variations of ambient dose equivalent

In Section 3.2, it was shown that the ambient neutron doses simulated with the MC model agree with the measurements within the uncertainties and thus that one can rely on the results of the simulations. In this subsection, the simulation results are used for analysis of the variation of the neutron ambient dose equivalent H*(10) as a function of position inside the room and treatment plan parameters. The simulations with the geometrical model without specifics of the room were used in order to make the results independent of the exact room geometry. The trends observed in the simulations are compared with those of the measurements performed at Skandion and, where available, also with the measurements performed at CCB IFJ PAN. All the results are presented in terms of ambient dose equivalent H*(10) per unit of absorbed dose in the center of the SOBP.

#### 3.3.1 Position

**Figure 8:**
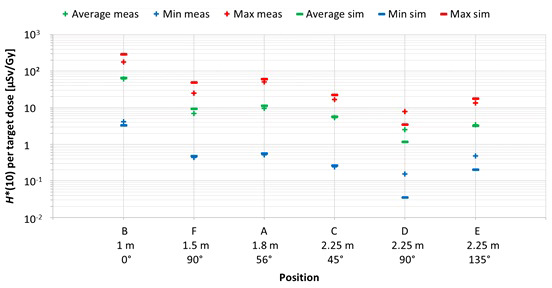
Plot of the variation of the simulated H*(10) values (bars) and the measured H*(10) values (crosses) at Skandion as a function of the position. Average (green), minimum (blue), and maximum (red) values are shown. The positions are ordered from left to right according to increasing distance from the isocenter and for same distances according to increasing angle with respect to the proton beam direction.

The first important factor influencing neutron H*(10) is the position inside the room. **Figure 8** shows the variation of the simulated H*(10) values (bars) and the measured H*(10) values (crosses) at Skandion as a function of the position. Average (green), minimum (blue), and maximum (red) values are shown. The positions are ordered from left to right according to increasing distance from the isocenter and for the same distances according to increasing angle with respect to the proton beam direction.

The observed trends for simulations and measurements are very similar. As expected, H*(10) decreases with increasing distance from the isocenter and H*(10) is lower in backward direction than in forward direction of the proton beam. H*(10) values between 0.04 µSv and 292 µSv per Gy target dose were obtained in the simulations. The highest doses were found for position B with an average overall treatment plan parameters of about 66 µSv per Gy. This was expected as it is the closest position at only 1 m from the isocenter and located in the forward direction of the beam. Lowest doses were found for positions D and E with on average over all treatment plan parameters of about 1 and 3 µSv per Gy, respectively. Also, this was expected as these positions are furthest away at 2.25 m distance in directions perpendicular and backward with respect to the proton beam direction for positions D and E, respectively. Finally, it can be seen that the measurements at position D are significantly higher than the simulations. This is probably due to the missing scattered neutrons from the room in the simulations as discussed in Section 3.2.2.

#### 3.3.2 Field size

**Figure 9:**
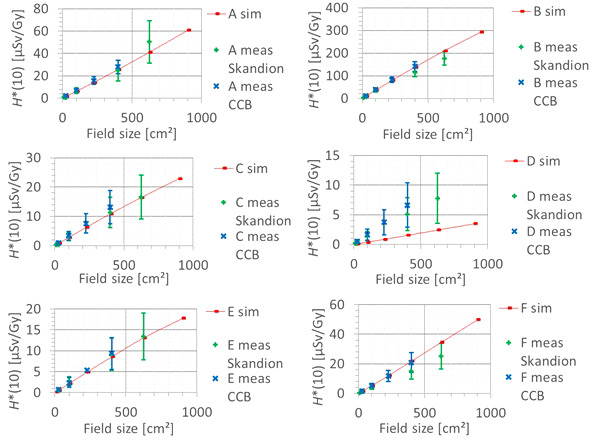
Plots of the variation of the simulated H*(10) (red bars) and measured H*(10) (green and blue crosses) as a function of the field size for a range of 15 cm and modulation width of 10 cm without a range shifter for the different positions.

**Figure 10:**
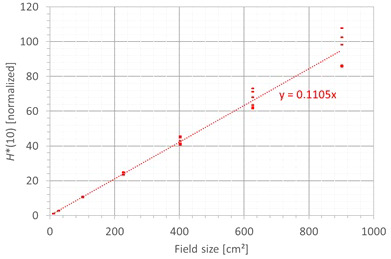
Plot of the variation of the simulated H*(10) as a function of the field size for all positions for a range of 15 cm and a modulation width of 10 cm without a range shifter with a linear fit through all the data points.

The second factor influencing neutron H*(10) is the field size. **Figure 9** shows the variation of the simulated H*(10) (red bars connected with lines) and measured H*(10) (green and blue crosses) as a function of the field size for a range of 15 cm and a modulation width of 10 cm without a range shifter for the different positions inside the room. The error bars on the measurement data points represent the *k* = 1 measurement uncertainties. **Figure 10** shows the same simulation data but for all positions in one plot together with a linear fit through all data points. In this plot, the H*(10) is for each position normalized to the H*(10) for the lowest field size for that position. The fit has no physical meaning, but can be used as an approximate scaling law for modeling the dependence of the neutron H*(10) on the field size.

The observed trends in simulations and measurements at Skandion and CCB are very similar. Only for position D are the measurements significantly higher than the simulations. This is probably due to the missing scattered neutrons from the room in the simulations as discussed in Section 3.2.2. The neutron H*(10) increases linearly with field size area for all positions. The H*(10) increases with a factor of about 100 when changing from a field size of 3 cm × 3 cm to a field size of 30 cm × 30 cm. Inside the phantom, the treatment volume is closer and the neutron source deviates more strongly from a distant point source. Therefore, inside the phantom, there might be a deviation from this linearity with an additional position dependence.

The simulations also showed that the neutron energy spectrum does not vary significantly with changes in the field size area.

#### 3.3.3 Range

**Figure 11:**
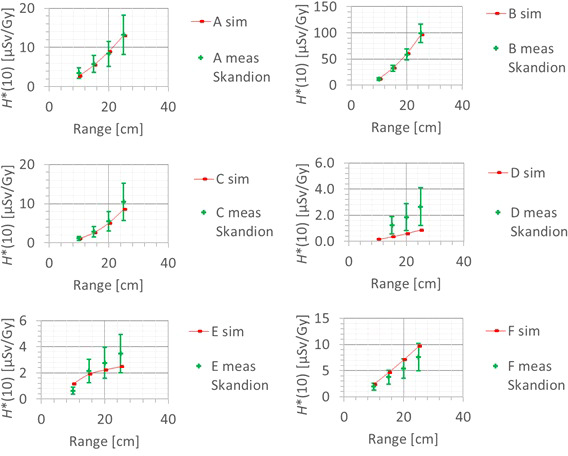
Plots of the variation of the simulated H*(10) (red bars) and measured H*(10) (green crosses) as a function of the range for a field size of 10 cm by 10 cm and a modulation width of 5 cm without a range shifter for the different positions.

**Figure 12:**
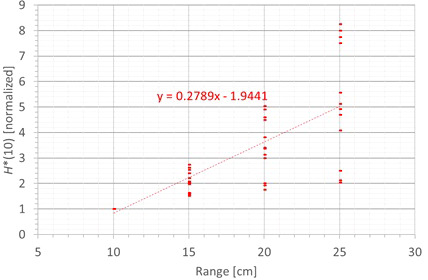
Plot of the variation of the simulated H*(10) as a function of the range for all modulation widths and positions for a field size of 10 cm by 10 cm without a range shifter with a linear fit through all the data points.

The next factor influencing the neutron H*(10) is the proton range and the corresponding proton energy. **Figure 11** shows the variation of the simulated H*(10) (red bars connected with lines) and measured H*(10) (green crosses) as a function of the range for a field size of 10 cm by 10 cm and a modulation width of 5 cm without a range shifter for the different positions. The error bars on the measurement data points represent the *k* = 1 measurement uncertainties. **Figure 12** shows the same simulation data for all positions in one plot together with the data for the other modulation widths and a linear fit through all data points. In this plot, the H*(10) is for each position and modulation width normalized to the H*(10) for the lowest range for that position and modulation width. The fit has no physical meaning, but can be used as an approximate scaling law for modeling the dependence of the neutron H*(10) on the proton range.

The observed trends in the simulations and measurements at Skandion are very similar. Only for position D are the measurements significantly higher than the simulations. This is probably due to the missing scattered neutrons from the room in the simulations as discussed in Section 3.2.2. For the 10-cm modulation width, there are also measurements performed at CCB that are very well in line with the measurements at Skandion and the simulations. It can be seen that the H*(10) increases relatively linearly with the range. In this case, a general linear scaling law does not reproduce the trends for all modulation widths and positions properly. The increase in H*(10) as a function of the range depends significantly on the position. This is probably related to the difference in distance to the treatment volume as the main neutron source for different positions. When changing the range from 10 cm to 25 cm, the H*(10) increases with a factor of two to eight, depending on the position and modulation width.

The simulations also showed that the neutron fluence energy spectrum has a shift of the high-energy neutron peak towards higher energies and a decrease of the thermal neutron contribution with an increase in range. This is expected because an increased range means increased proton energy and thus also an increase of the energy of the high-energy neutrons and a decrease of the fraction of thermalized neutrons.

#### 3.3.4 Modulation width

**Figure 13:**
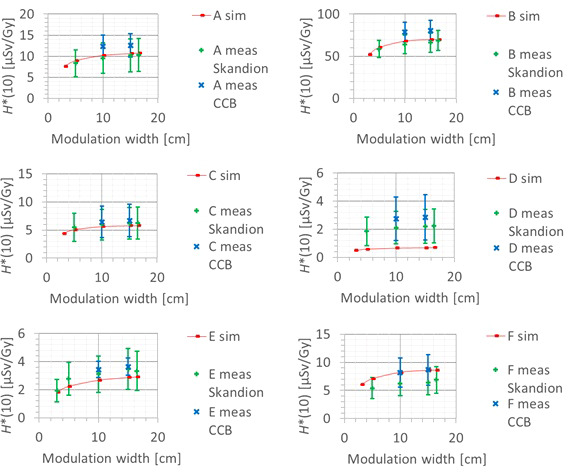
Plots of the variation of the simulated H*(10) (red bars) and measured H*(10) (green and blue crosses) as a function of the modulation width for a range of 20 cm and a field size of 10 cm by 10 cm without a range shifter for the different positions.

**Figure 14:**
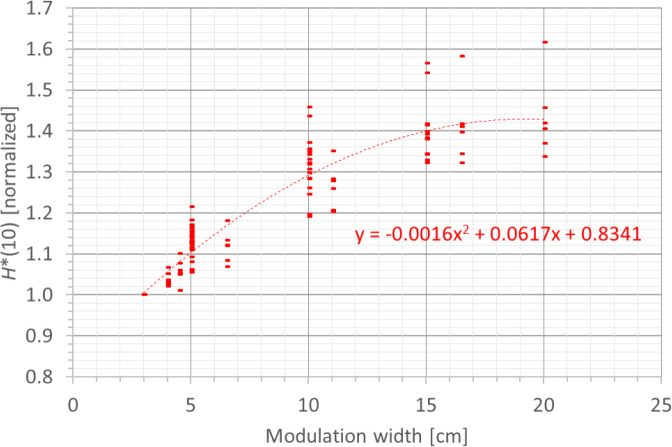
Plot of the variation of the simulated H*(10) as a function of the modulation width for all ranges and positions for a field size of 10 cm by 10 cm without a range shifter with a quadratic fit through all the data points.

A fourth factor influencing the neutron H*(10) is the modulation width. **Figure 13** shows the variation of the simulated H*(10) (red bars connected with lines) and measured H*(10) (green and blue crosses) as a function of the modulation width for a range of 20 cm and a field size of 10 cm by 10 cm without a range shifter for the different positions. The error bars on the measurement data points represent the *k* = 1 measurement uncertainties. **Figure 14** shows the same simulation data for all positions in one plot together with the data for the other ranges and a quadratic fit through all data points. In this plot, the H*(10) is for each position and range normalized to the H*(10) for the lowest modulation width for that position and range. The fit has no physical meaning, but can be used as an approximate scaling law for modeling the dependence of the neutron H*(10) on the modulation width.

The observed trends in the simulations and measurements at Skandion and CCB are very similar. Only for position D are the measurements significantly higher than the simulations. This is probably due to the missing scattered neutrons from the room in the simulations as discussed in Section 3.2.2. It can be seen that the H*(10) increases quadratically with the modulation width and that the increase becomes less steep for higher modulation widths. A quadratic fit reproduces the trends for different ranges and positions quite well, but there is a limited dependence on range and position. Similar to the range, this is probably related to the difference in distance to the treatment volume as the main neutron source for different positions. The H*(10) increases between 35% and 60% when increasing the modulation width from 3 cm to 20 cm, depending on the range and position.

The simulations also showed that the neutron energy spectrum does not change significantly when changing the modulation width.

#### 3.3.5 Range shifter and air gap

The last investigated factor influencing the neutron H*(10) is the use of a range shifter. It was found both in the measurements and the simulations that the H*(10) increases on average by a factor of about two when introducing a range shifter for the same treatment. The increase was higher for lower ranges. For all positions except for position D, the increase is limited to a factor of 2.5, while for position D, an increase up to a factor of 6 was observed. This is probably due to the fact that the range shifter is positioned within line of sight from position D. The absorbed dose measured at position A with the Sievert was also shown to increase up to a factor of nine when introducing a range shifter. This increase is probably not due to neutrons but due to protons from the range shifter as discussed in detail in (23).

These observations are within expectations. When performing the same treatment, introducing a range shifter means increasing the proton energies as can be seen in **Table A.1** in the appendix. This leads to an additional neutron creation in the range shifter. As a first approximation, one can consider that the range is increased by the solid water equivalent thickness of the range shifter. It can be seen indeed that the H*(10) values simulated with the range shifter are close to the H*(10) values simulated without a range shifter for a range corresponding to the sum of the range with the range shifter and the solid water equivalent thickness of the range shifter.

The simulations also showed that, similar to an increase of the range, the use of a range shifter leads to a shift of the high-energy neutron peak towards higher energies and a decrease of the thermal neutron contribution.

**Figure 15:**
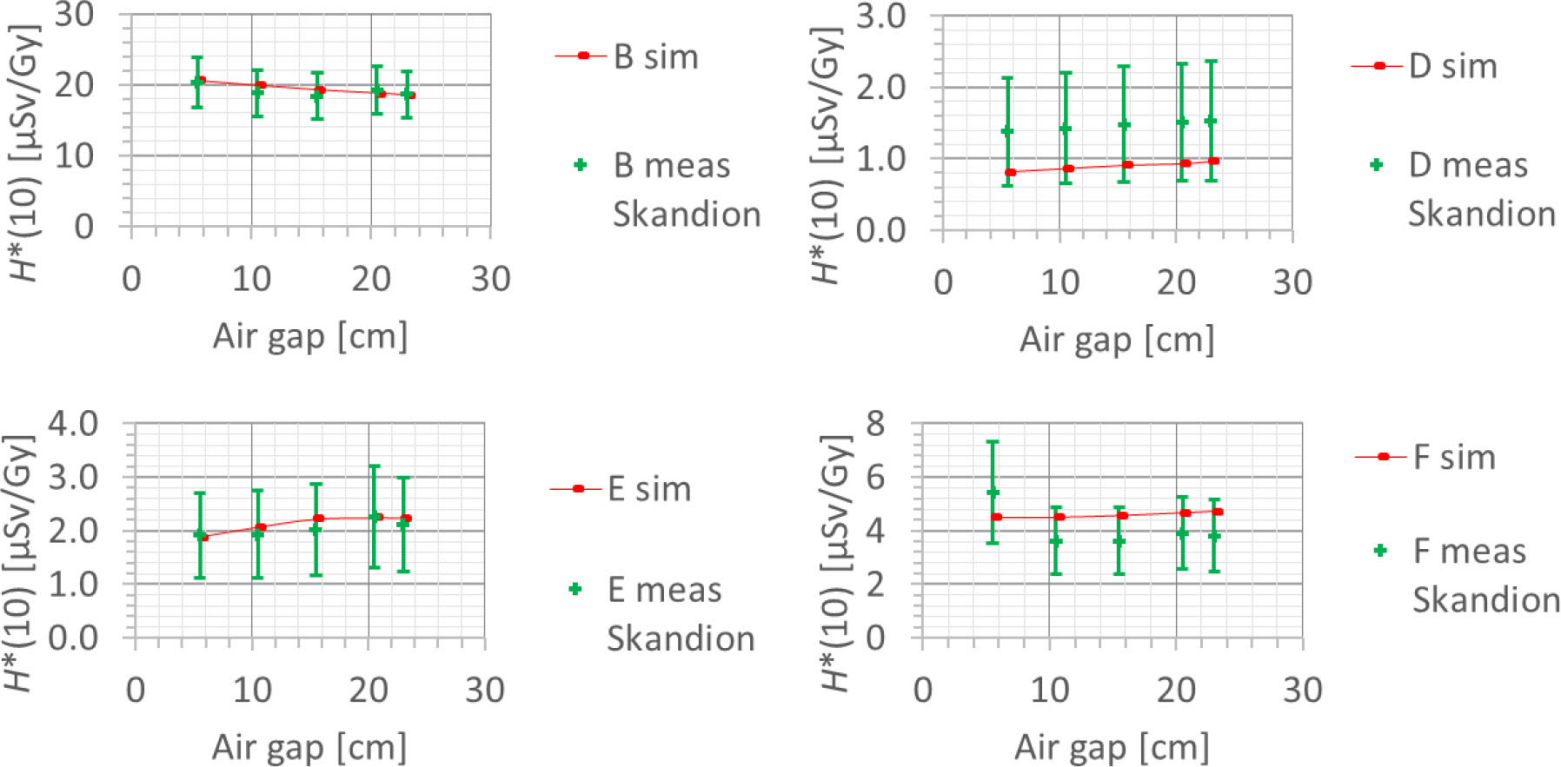
Plots of the variation of the simulated H*(10) (red bars) and measured H*(10) (green crosses) as a function of the air gap between the range shifter and the phantom for a range of 10 cm, a modulation width of 5 cm, and a field size of 10 cm by 10 cm for the different positions.

Also, the dependence of the neutron H*(10) on the air gap between the range shifter and the phantom was studied, in both the simulations and the measurements. The error bars on the measurement data points represent the *k* = 1 measurement uncertainties. **Figure 15** shows the variation of the simulated H*(10) (red bars connected with lines) and measured H*(10) (green crosses) as a function of the air gap between the range shifter and the phantom for a range of 10 cm, a modulation width of 5 cm, and a field size of 10 cm × 10 cm for the different positions. Both the simulations and the measurements at Skandion showed no significant effect of the variation of the air gap on the neutron H*(10) for the investigated positions. Further research is necessary to evaluate the potential influence of the air gap at other positions such as inside the phantom, closer to the range shifter. The absorbed dose measured at position A with the Sievert was shown to increase with increasing air gap. This increase is probably not due to neutrons but due to protons from the range shifter as discussed in detail in (23).

## 4 Conclusions and outlook

A general MC radiation transport model was set up for simulation of neutron doses from scattered and secondary radiation at PBS proton therapy facilities. This model was successfully verified by comparison of simulated and measured ambient neutron doses for the irradiations of a solid water phantom at two different PBS proton therapy facilities using a variety of treatment plans. The simulated SOBP profile properly reflected the range and modulation width of the plans. Furthermore, the deviations between the simulated neutron H*(10) values and ambient neutron monitor measurements at six different positions around the solid water phantom were well within the expected uncertainties. Finally, the simulated neutron fluence energy spectra were in good agreement with Bonner sphere measurements performed during a previous measurement campaign with a similar setup. For the positions close to the phantom where scattered neutrons from the room do not contribute significantly to the dose, the agreement between simulated and measured neutron H*(10) was within 30%. Therefore, it is expected that with this general MC radiation transport model of PBS proton therapy facilities, it will also be possible to simulate out-of-field doses inside the phantom with a similarly good accuracy.

The MC simulations facilitated a detailed study of the variation of neutron H*(10) with position inside the room, field size, range, modulation width, use of a range shifter, and air gap between the range shifter and the phantom. The neutron H*(10) depends strongly on the position inside the room with a general decrease of the neutron H*(10) with increasing distance from the isocenter and higher neutron H*(10) in the forward direction of the proton beam in comparison with the backward direction. The linear increase with field size and the increase of up to a factor of eight with increasing range were found to be the strongest influences on the neutron H*(10). The neutron H*(10) was also found to increase by up to about 60% with increasing modulation width. The use of a range shifter on average increases the H*(10) by a factor of two. The air gap between the range shifter and the phantom did not have a significant influence on the neutron H*(10) at the investigated positions. Further research is needed to evaluate potential influence of the air gap inside the phantom, closer to the range shifter. Furthermore, it was found that the variations of the neutron H*(10) with the treatment plan parameters have interdependencies and also depend on the position inside the room. This inhibits the use of simple scaling factors to predict the H*(10) more precisely than within a factor of about three close to the phantom. More precise prediction of the neutron H*(10) requires simulations at the location of interest. Finally, this work demonstrates that, when reporting on out-of-field neutron doses in proton therapy, it is important not only to normalize the out-of-field neutron doses to the target dose or product of target dose with treatment volume, but also to provide the treatment plan parameters. This is crucial in order to be able to compare results from different studies.

The ambient neutron doses per unit of target dose in this study varied between 3 µSv/Gy and 300 µSv/Gy at 1 m from the isocenter in the beam direction and between 0.5 µSv/Gy and 50 µSv/Gy at 1.5 m from the isocenter perpendicular to the beam direction. The out-of-field neutron dose for a specific treatment in an organ at a certain distance from the isocenter can already be roughly estimated based on the dose data and scaling laws provided in this work in combination with the inverse square distance law to very roughly model the dependence on the distance from the isocenter. As a first test, this was done for the recently published extensive study of out-of-field neutron doses for a proton therapy brain treatment (24). The brain treatment in (24) can be approximated by an irradiation with 33 cm² field size, 5 cm modulation width, and 18 cm range. The irradiations in this treatment were performed under three different angles. The out-of-field neutron doses per unit of target dose assessed in (24) by means of measurements and MC radiation transport simulations ranged between 10 µSv/Gy at 50 cm from the isocenter and 1,000 µSv/Gy at 10 cm from the isocenter. The most comparable dose data from this work are the dose data for position B (in beam direction at 1 m from the isocenter) and position F (perpendicular to beam direction at 1.5 m from the isocenter) for the irradiation with 20 cm range, 5 cm modulation width, and 100 cm² field size. Based on the scaling laws obtained in this work, these dose data were divided by a factor of three to correct for the larger field size and multiplied with a factor of 0.84 to correct for the larger range. Then, they were recalculated to the distances of 10 cm and 50 cm by means of the inverse square distance law. This resulted in doses of 70 µSv/Gy (in beam direction) and 10 µSv/Gy (perpendicular to beam direction) at 50 cm and 1700 µSv/Gy (in beam direction) and 300 µSv/Gy (perpendicular to beam direction) at 10 cm. These doses are very well in line with the 10 µSv/Gy at 50 cm and 1000 µSv/Gy at 10 cm obtained in (24). This example demonstrates that rough estimates of out-of-field neutron doses can already be obtained easily based on the ambient neutron dose data and scaling laws provided in this work.

More accurate assessment of out-of-field neutron doses requires a more sophisticated approach that will be explored in future work. It is planned to use this verified MC radiation transport model for PBS proton therapy facilities to directly simulate out-of-field neutron doses inside the phantom instead of ambient neutron doses outside the phantom. The phantom will be filled with 2-mm-sized boxes to allow high spatial resolution close to the field where large dose gradients can be expected. Fluence energy spectra will be tallied in these boxes for neutrons, as well as for protons and photons that can also contribute significantly to the out-of-field doses. These fluence energy spectra will then be convoluted with fluence to dose equivalent conversion coefficients from literature to obtain the out-of-field doses per unit of target dose in terms of dose equivalent. The updated simulation model will first be verified again for a few specific sets of treatment parameters by comparison with a previous in-phantom measurement campaign performed within EURADOS WG9 at the Trento proton therapy facility. After this verification, the simulations will be performed for a large series of relevant field sizes, ranges, modulation widths, and range shifters. The simulations will be performed both with beam direction along the long and the short axis of the phantom. The simulated out-of-field doses will then be used to build a library and a corresponding look-up tool to allow assessment of the out-of-field doses at PBS proton therapy facilities as a function of treatment plan parameters and position with respect to the isocenter and beam direction. The rectangular field treatment plans and the solid water phantom used in these simulations are of course simplified in comparison with the actual treatment of a real patient. However, actual treatment plans can be simplified as one or a combination of several rectangular treatment plans. In the first instance, different organs can be approximated as a series of boxes in the solid water phantom at a representative distance and angle with respect to the isocenter and beam direction. Later on, the simulations in the solid water phantom can be made more realistic by performing simulations in a series of representative anthropomorphic phantoms including the actual organs. In this way, the library and tool can be extended continuously and be made more realistic over time to allow, for instance, also taking into account differences between pediatric, adult, and pregnant patients. The tool that will be developed in this way will enable the optimization of treatment planning in terms of out-of-field doses and associated detrimental effects on healthy tissue.

## Data availability statement

The original contributions presented in the study are included in the article/supplementary material. Further inquiries can be directed to the corresponding author.

## Author contributions

OV: conceptualization, experimental setup, analysis of measurement data, Monte Carlo simulation design, setup, execution and analysis, manuscript writing, review and editing. LS: conceptualization, experimental design and setup, analysis of measurement data, manuscript writing, review and editing. JL: experimental setup, analysis of measurement data, manuscript writing, review and editing. LE: experimental setup, analysis of measurement data, manuscript review and editing. NM: conceptualization, experimental design and setup, analysis of measurement data. ML: experimental design and setup. AA: experimental design and setup, manuscript review. VM: conceptualization, experimental design and setup, analysis of measurement data, manuscript review and editing. FT: experimental setup, analysis of measurement data, manuscript review and editing. ST: experimental setup, analysis of measurement data. IM-R: experimental setup, analysis of measurement data, manuscript review and editing. CD: experimental setup, analysis of measurement data. MR-E: experimental setup, analysis of measurement data. PO: experimental setup, analysis of measurement data. RH: conceptualization, experimental design, manuscript review and editing. PO: conceptualization, experimental design, experimental setup, manuscript review and editing. All authors contributed to the article and approved the submitted version.

## Funding

This project has received funding from INSPIRE from the European Union’s Horizon 2020 research and innovation programme under grant agreement No (730983) IM-R acknowledges the financial support from the Spanish Ministry of Science, Innovation and Universities (RYC2018-024043-I) The work of Ondrej Ploc on the paper was funded by EU Operational Program Research, Development, and Education, call 02_15_003 in project CRREAT, number CZ.02.1.01/0.0/0.0/15_003/0000481.

## Conflict of interest

The authors declare that the research was conducted in the absence of any commercial or financial relationships that could be construed as a potential conflict of interest.

## Publisher’s note

All claims expressed in this article are solely those of the authors and do not necessarily represent those of their affiliated organizations, or those of the publisher, the editors and the reviewers. Any product that may be evaluated in this article, or claim that may be made by its manufacturer, is not guaranteed or endorsed by the publisher.
