# Supplementary material for: Simulation and experimental verification of ambient neutron doses in a pencil beam scanning proton therapy room as a function of treatment plan parameters
Source: Front Oncol. 2022 Sep 8;12:903537. doi: 10.3389/fonc.2022.903537 (PMC9494550; doi:10.3389/fonc.2022.903537)
Supplement: Supplementary file 1 [file Table_1.docx]

*Table A.1: Overview of all irradiations and their treatment plan parameters for the measurement campaign performed at the Skandion proton therapy facility.*

| **Irradiation number** | **Range [cm]** | **Modulation width [cm]** | **Field size [cm]** | **Range shifter** | **Air gap [cm]** | **Minimal proton energy [MeV]** | **Maximal proton energy [MeV]** |
| --- | --- | --- | --- | --- | --- | --- | --- |
| 1 | 8 | 3 | 10 | no | - | 80 | 103 |
| 2 | 8 | 4 | 10 | no | - | 71 | 103 |
| 3 | 8 | 4.5 | 10 | no | - | 66 | 103 |
| 4 | 10 | 3 | 10 | no | - | 96 | 117 |
| 5 | 10 | 5 | 10 | no | - | 80 | 117 |
| 6 | 10 | 6.5 | 10 | no | - | 66 | 117 |
| 7 | 15 | 3 | 10 | no | - | 130 | 146 |
| 8 | 15 | 5 | 10 | no | - | 117 | 146 |
| 9 | 15 | 10 | 10 | no | - | 80 | 146 |
| 10 | 15 | 11.5 | 10 | no | - | 66 | 146 |
| 11 | 20 | 3 | 10 | no | - | 160 | 173 |
| 12 | 20 | 5 | 10 | no | - | 149 | 173 |
| 13 | 20 | 10 | 10 | no | - | 119 | 173 |
| 14 | 20 | 15 | 10 | no | - | 80 | 173 |
| 15 | 20 | 16.5 | 10 | no | - | 65 | 173 |
| 16 | 25 | 3 | 10 | no | - | 184 | 197 |
| 17 | 25 | 5 | 10 | no | - | 175 | 197 |
| 18 | 25 | 10 | 10 | no | - | 148 | 197 |
| 19 | 25 | 15 | 10 | no | - | 118 | 197 |
| 20 | 25 | 20 | 10 | no | - | 81 | 197 |
| 21 | 15 | 10 | 3 | no | - | 80 | 146 |
| 22 | 15 | 10 | 5 | no | - | 80 | 146 |
| 23 | 15 | 10 | 20 | no | - | 80 | 146 |
| 24 | 15 | 10 | 25 | no | - | 80 | 146 |
| 25 | 15 | 10 | 30 | no | - | 80 | 147 |
| 26 | 10 | 3 | 10 | yes | 23 | 122 | 138 |
| 27 | 10 | 5 | 10 | yes | 5.5 | 107 | 138 |
| 28 | 10 | 5 | 10 | yes | 10.5 | 107 | 138 |
| 29 | 10 | 5 | 10 | yes | 15.5 | 107 | 138 |
| 30 | 10 | 5 | 10 | yes | 20.5 | 107 | 138 |
| 31 | 10 | 5 | 10 | yes | 23 | 107 | 138 |
| 32 | 10 | 7 | 10 | yes | 23 | 92 | 138 |
| 33 | 10 | 10 | 10 | yes | 23 | 68 | 138 |
| 34 | 15 | 3 | 10 | yes | 23 | 150 | 165 |
| 35 | 15 | 5 | 10 | yes | 23 | 150 | 165 |
| 36 | 15 | 10 | 10 | yes | 5.5 | 108 | 165 |
| 37 | 15 | 10 | 10 | yes | 10.5 | 108 | 165 |
| 38 | 15 | 10 | 10 | yes | 15.5 | 108 | 165 |
| 39 | 15 | 10 | 10 | yes | 20.5 | 108 | 165 |
| 40 | 15 | 10 | 10 | yes | 23 | 108 | 165 |
| 41 | 15 | 15 | 10 | yes | 23 | 66 | 165 |
| 42 | 25 | 3 | 10 | yes | 23 | 200 | 212 |
| 43 | 25 | 5 | 10 | yes | 23 | 191 | 212 |
| 44 | 25 | 10 | 10 | yes | 5.5 | 166 | 212 |
| 45 | 25 | 10 | 10 | yes | 10.5 | 166 | 212 |
| 46 | 25 | 10 | 10 | yes | 15.5 | 166 | 212 |
| 47 | 25 | 10 | 10 | yes | 20.5 | 166 | 212 |
| 48 | 25 | 10 | 10 | yes | 23 | 166 | 212 |
| 49 | 25 | 15 | 10 | yes | 23 | 139 | 212 |
| 50 | 25 | 20 | 10 | yes | 23 | 108 | 212 |
| 51 | 25 | 25 | 10 | yes | 23 | 67 | 212 |
