# Supplementary material for: Simulation and experimental verification of ambient neutron doses in a pencil beam scanning proton therapy room as a function of treatment plan parameters
Source: Front Oncol. 2022 Sep 8;12:903537. doi: 10.3389/fonc.2022.903537 (PMC9494550; doi:10.3389/fonc.2022.903537)
Supplement: Supplementary file 2 [file Table_2.docx]

*Table A.2: Overview of all irradiations and their treatment plan parameters for the measurement campaign performed at the IFJ PAN CCB proton therapy facility.*

| **Irradiation number** | **Range [cm]** | **Modulation width [cm]** | **Field size [cm]** | **Range shifter** | **Air gap [cm]** | **Minimal proton energy [MeV]** | **Maximal proton energy [MeV]** |
| --- | --- | --- | --- | --- | --- | --- | --- |
| 1 | 10 | 10 | 10 | no | - | 75 | 141 |
| 2 | 15 | 10 | 10 | no | - | 81 | 147 |
| 3 | 15 | 10 | 15 | no | - | 81 | 147 |
| 4 | 15 | 10 | 20 | no | - | 81 | 147 |
| 5 | 15 | 10 | 5 | no | - | 81 | 147 |
| 6 | 20 | 10 | 10 | no | - | 118 | 173 |
| 7 | 25 | 10 | 10 | no | - | 148 | 197 |
| 8 | 30 | 10 | 10 | no | - | 174 | 219 |
| 9 | 15 | 15 | 10 | no | - | 74 | 169 |
| 10 | 20 | 15 | 10 | no | - | 81 | 173 |
| 11 | 20 | 20 | 10 | no | - | 74 | 193 |
